# Etifoxine, a TSPO Ligand, Worsens Hepatitis C-Related Insulin Resistance but Relieves Lipid Accumulation

**DOI:** 10.1155/2019/3102414

**Published:** 2019-03-11

**Authors:** Yu-Min Lin, Hung-Yu Sun, Wen-Tai Chiu, Hui-Chen Su, Yu-Chieh Chien, Heng-Ai Chang, Lee-Won Chong, Hung-Chuen Chang, Kung-Chia Young, Chyi-Huey Bai, Chiung-Wen Tsao

**Affiliations:** ^1^Department of Gastroenterology, Shin Kong Wu Ho-Su Memorial Hospital, Taipei 11101, Taiwan; ^2^School of Medicine, Fu Jen Catholic University, New Taipei City 24205, Taiwan; ^3^Department of Biomedical Engineering, College of Biology, Hunan University, Changsha, Hunan 410082, China; ^4^Department of Medical Laboratory Science and Biotechnology, College of Medicine, National Cheng Kung University, Tainan 70101, Taiwan; ^5^Department of Biomedical Engineering, College of Engineering, National Cheng Kung University, Tainan 70101, Taiwan; ^6^Department of Pharmacy, Chi-Mei Medical Center, Tainan 71004, Taiwan; ^7^Department of Nursing, Chung Hwa University of Medical Technology, Tainan 71703, Taiwan; ^8^Institute of Basic Medical Sciences, National Cheng Kung University, Tainan 70101, Taiwan; ^9^Department of Public Health, College of Medicine, Taipei Medical University, Taipei 11031, Taiwan

## Abstract

Etifoxine, an 18 kDa translocator protein (TSPO) agonist for the treatment of anxiety disorders in clinic, may be able to cause acute liver injury or cytolytic hepatitis. TSPO has been demonstrated to participate in inflammatory responses in infective diseases as well as to modulate glucose and lipid homeostasis. Hepatitis C virus (HCV) infection disrupts glucose and lipid homoeostasis, leading to insulin resistance (IR). Whether TSPO affects the HCV-induced IR remains unclear. Here, we found that the administration of etifoxine increased the TSPO protein expression and recovered the HCV-mediated lower mitochondrial membrane potential (MMP) without affecting HCV infection. Moreover, etifoxine reversed the HCV-induced lipid accumulation by modulating the expressions of sterol regulatory element-binding protein-1 and apolipoprotein J. On the other hand, in infected cells pretreated with etifoxine, the insulin-mediated insulin receptor substrate-1/Akt signals, forkhead box protein O1 translocation, and glucose uptake were blocked. Taken together, our results pointed out that etifoxine relieved the HCV-retarded MMP and reduced the lipid accumulation but deteriorated the HCV-induced IR by interfering with insulin signal molecules.

## 1. Introduction

Hepatitis C virus (HCV) is the cause of a global health problem with approximately 3% of the world's population being chronically infected. HCV infection is also a major risk factor that can cause hepatic insulin resistance (IR), steatosis, fibrosis, cirrhosis, and hepatocellular carcinoma. Notably, clinical evidence indicates a link between HCV and IR, which is a common metabolic disorder in the prediabetic state. The frequency of diabetes is approximately 20% in patients with HCV [1]. HCV induces IR in a genotype-dependent fashion, contributing to steatosis, the progression of fibrosis, apoptosis, and resistance to interferon-*α* therapy [2]. The intricately pathological IR mechanisms in HCV core protein-infected hepatocytes are associated with an impaired activation of insulin receptor substrate (IRS)-1 and Akt and a blockade of insulin-stimulated glucose uptake [3]. Moreover, upregulation of sterol regulatory element-binding protein-1 (SREBP-1) and the interference with forkhead box protein O1 (FoxO1) transcriptional activity contribute to HCV core protein-induced IR [4]. In addition, mitochondrial dysfunction and oxidative stress are involved in the development of IR [2]. Nevertheless, the mechanism how HCV promotes IR has not yet been fully elucidated.

18 kDa translocator protein (TSPO), also named peripheral-type benzodiazepine receptor or mitochondrial benzodiazepine receptor, is prevalently expressed in immune cells and peripheral tissues [5–7]. TSPO is involved in a variety of biological processes [8–14]. In addition, altered TSPO expressions are related to cancer, neurodegenerative disorders, various forms of brain injury and inflammation, and anxiety [15–21]. Interestingly, few studies have focused on the regulation of TSPO in neuroinflammation of infective diseases [22–27]. However, whether TSPO directly regulates HCV infection or not is still unclear.

Growing evidence has shown that TSPO ligands modulate glucose homeostasis, lipid transport, and adipocyte functions [11, 28, 29]. TSPO may act as a potential therapeutic target due to its ability to increase macrophage cholesterol efflux to apolipoprotein (apo) acceptors involved in the reduction of macrophage neutral lipid mass and lipogenesis [11]. However, the precise mechanisms for this issue are still unclear.

In the present study, etifoxine as a TSPO agonist [30] was used to investigate the signal molecules related to HCV-induced IR, including IRS-1, Akt, FoxO1, SREBP-1, glucose uptake, lipid accumulation with altered apoJ expression, and mitochondrial membrane potential (MMP).

## 2. Materials and Methods

### 2.1. Chemicals

Etifoxine was purchased from Tocris Bioscience (Bristol, UK), and insulin was purchased from Sigma-Aldrich (St. Louis, MO, USA).

### 2.2. Antibodies

Antibodies against Akt (catalog No. 4685), FoxO1 (catalog No. 2880), and lamin A (catalog No. 4777) were purchased from Cell Signaling Technology, Inc. (Danvers, MA, USA). Antibodies against HCV core (catalog No. ab2740), NS3 (catalog No. ab65407), SREBP-1 (catalog No. ab28481), apoJ/clusterin (catalog No. ab69644), and TSPO (catalog No. ab109497) were purchased from abcam (Cambridge, UK). Antibody against IRS-1 (catalog No. 06-248) was purchased from EMD Millipore (Billerica, MA, USA). Antibody against phospho-IRS-1 (Tyr896) (catalog No. 1813-1) was purchased from Epitomics (Burlingame, CA, USA). Antibody against phospho-Akt (Ser473) (catalog No. GTX61708) was purchased from GeneTex, Inc. (Irvine, CA, USA). Antibody against *β*-actin (catalog No. A5441) was purchased from Sigma-Aldrich.

### 2.3. Cell Culture

The human hepatocellular carcinoma cells, Huh7.5, that carry nonstructural protein (NS)3/4A protease-based secreted alkaline phosphatase (SEAP) reporter were cultured in Dulbecco's Modified Eagle's Medium (Sigma-Aldrich) containing 2 *μ*g/ml blasticidin (Life Technologies, Grand Island, NY, USA).

### 2.4. Evaluation of HCV Infection Using NS 3/4A Protease-Based SEAP Reporter Assay

The Huh7.5-SEAP cells were inoculated with HCV at the multiplicity of infection (MOI) of 0.01 and a commercial Phospha-Light assay kit (Invitrogen, Carlsbad, CA, USA) was applied to determine the SEAP activity in the culture media at the indicated time. Please refer to our previous work for the detail protocol [31, 32]. Manufacture of HCV infection system was approved by Institutional Biosafety Committee, Chung Hwa University of Medical Technology.

### 2.5. Cell Viability

The viability of cells was determined using a commercial 3-(4,5-dimethylthiazol-2-yl)-5-(3-carboxymethoxyphenyl)-2-(4-sulfophenyl)-2H-tetrazolium (MTS) assay kit. The details are shown in [32].

### 2.6. MMP Measurement

Mitochondrial function was indirectly evaluated with the variation in MMP measured by JC-1 dye (MMP probe, Thermo Fisher Scientific Inc., Waltham, MA, USA) as previously described [33]. Cells were seeded in 96-well at 37°C overnight, and various concentrations of etifoxine were added at 37°C for 6 days. Carbonyl cyanide 3-chlorophenylhydrazone (CCCP, Sigma-Aldrich) acted as a positive control at 37°C for 2-h ahead of the end of the 6-day incubation time. After being washed with phosphate buffered saline (PBS), JC-1 dye was added to cells at 37°C for 30 min in a dark place. Subsequently, both red (optical density, OD 550-600 nm) and green (OD 485-535 nm) fluorescence emissions were analyzed. Data was calculated as the ratio of red fluorescence to green fluorescence.

### 2.7. Western Blot Analysis

The details are shown in [32]. Briefly, cells were lysed by lysis buffer containing protease inhibitor cocktail (Millipore, Burlington, MA, USA) and cell lysate was collected after centrifugation. Proteins were separated in sodium dodecyl sulfate-polyacrylamide gel, transferred to a polyvinylidene difluoride membrane (Millipore), blocked with skim milk, and probed with primary antibodies. The protein expressions were evaluated using horseradish peroxidase-conjugated secondary antibodies via enhanced chemiluminescence reagent (GE, Little Chalfont, UK) and analyzed with LAS4000 software (GE).

### 2.8. Nuclear Extraction

The commercially available CHEMICON® nuclear extraction kit (Millipore) was used according to the manufacturer's protocols.

### 2.9. Immunofluorescence Analysis

The immunofluorescence analysis was performed as previously described [32]. Briefly, cells were fixed with 4% paraformaldehyde, permeabilized with 0.2% Triton X-100, blocked with bovine serum albumin/PBS. The target proteins, including HCV core and SREBP1, were stained with corresponding antibodies and then visualized using Alexa Fluor conjugated secondary antibodies.

For observation of neutral lipid morphology, the lipid droplets and nucleus were stained with fluorescent neutral lipid dye 4,4-difluoro-1,3,5,7,8-pentamethyl-4-bora-3a,4a-diaza-s-indacene (BODIPY 493/503, Invitrogen) and 4,6-diamidino-2-phenylindole (DAPI) (Sigma-Aldrich), respectively. The image data was collected using confocal laser scanning microscope (Olympus FluoView, FV1000, Shinjuku-ku, Tokyo, Japan).

### 2.10. Glucose Uptake

A commercially available glucose uptake cell-based assay kit (Cayman Chemical Co., Ann Arbor, MI, USA) was used according to the manufacturer's protocols.

### 2.11. Lipid Accumulation

Intracellular lipid content was evaluated by oil red O staining (Sigma-Aldrich) as previously described [32].

### 2.12. Statistics

Mean and standard deviation (SD) were expressed. Two independent samples* t*-test or one-way analysis of variances were used to compare the differences between the two groups. An alpha of 0.05 was used as the cutoff for significance.

## 3. Results

### 3.1. Etifoxine Rescued the HCV-Disturbed MMP Without Modifying HCV Infection

**Figure 1:**
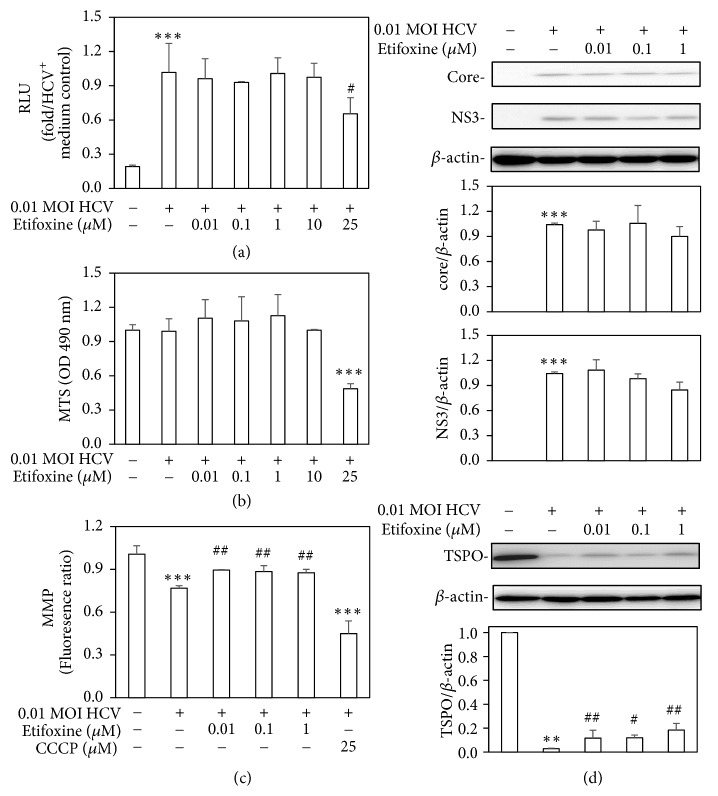
*Effects of etifoxine on SEAP activity, cell viability, MMP, and viral and TSPO protein levels.* (a) Huh7.5-SEAP cells (4 x 10^4^) were infected with 0.01 MOI of HCV and treated with etifoxine (0.01-25 *μ*M) in a 24-well plate for 6 days after treatment. Supernatants were collected for determining SEAP activity as a reflection of the quantitative evaluation of HCV infection. Data were analyzed by plotting the signal obtained as relative light unit (RLU) in which the HCV positive control is set to one-fold. (b) Cell viability obtained from 8 × 10^3^ cells/group was examined with a commercial MTS assay kit. (c) Huh7.5-SEAP cells (8 × 10^3^) infected with HCV were seeded in a 96-well plate and added with etifoxine (0.01-1 *μ*M) for 6-day incubation or 25 *μ*M of CCCP for 2 h. After cells were added with JC-1 dye and incubated for 30 min in dark place, data was calculated with the ratio of red fluorescence divided by green fluorescence. (d) Cell lysates (7 × 10^5^) were collected and the core, NS3, TSPO, and *β*-actin levels were detected by Western blot. Data are expressed as mean ±SD obtained from three individual experiments. ^*∗∗*^*p* < 0.01 and ^*∗∗∗*^*p* < 0.001 vs. the medium control group; ^#^*p* < 0.05 and ^##^*p* < 0.01 vs. the HCV-infected Huh 7.5-SEAP group.

The effects of the TSPO agonist, etifoxine, on HCV infection were first investigated. The Huh7.5-SEAP cells were infected with HCV at a MOI of 0.01 in the presence of etifoxine (0.01-25 *μ*M). The SEAP activity as a reflection of the quantitative evaluation of HCV infection in culture media was determined at 6-day post-infection. Without inducing cell cytoxicity, etifoxine at <10 *μ*M did not significantly affect SEAP activity (Figures 1(a) and 1(b)).

Since TSPO regulates mitochondrial functions [10], the effects of etifoxine on MMP were also investigated under HCV infection. A lower level of fluorescence ratio in Huh7.5-SEAP cells infected with HCV was observed, indicating that HCV disrupted MMP on day 6 after infection. The CCCP at 25 *μ*M acted as a positive control for the attenuation of MMP. Etifoxine at a low concentration of 0.01-1 *μ*M was able to reverse the HCV-induced lower MMP in Huh7.5-SEAP cells (Figure 1(c)). Although etifoxine at 0.01-1 *μ*M did not directly affect the viral core and NS3 protein levels, it partially activated TSPO expression in HCV infection as determined by Western blot (Figure 1(d)).

### 3.2. Etifoxine Aggravated the HCV-Reduced Activations of IRS-1 and Akt

**Figure 2:**
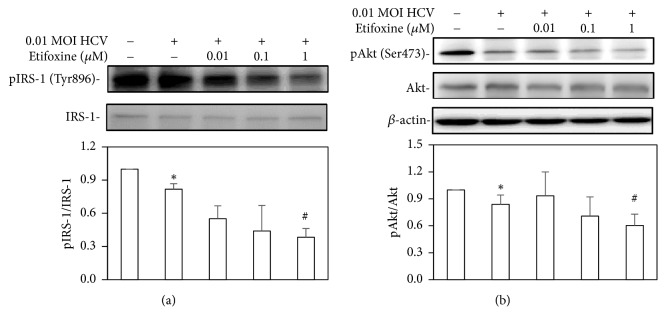
*Effects of etifoxine on IRS-1 and Akt phosphorylation.* (a and b) Huh7.5-SEAP cells (7 × 10^5^) were infected with 0.01 MOI of HCV and treated with etifoxine (0.01-1 *μ*M) in a 6-well plate for 6 days after treatment. Cell lysates were collected and the phospho-IRS-1 (Tyr896), IRS-1, phospho-Akt (Ser473), Akt, and *β*-actin levels were detected by Western blot. Data are expressed as mean ±SD obtained from three individual experiments. ^*∗*^*p* < 0.05 vs. the medium control group; ^#^*p* < 0.05 vs. the HCV-infected Huh 7.5-SEAP group.

To clarify the effects of etifoxine on HCV-induced IR, the insulin-mediated signal molecules were also examined in HCV-infected cells. The phosphorylation motifs of IRS-1 on Tyr896 and Akt on Ser473, which mediate and activate the metabolic functions of insulin, were therefore evaluated by Western blot analysis. HCV-infected Huh7.5-SEAP cells had the decreased phosphorylated levels of IRS-1 on Tyr896 and of Akt on Ser473 (Figures 2(a) and 2(b)). Addition of etifoxine increased the decline of phosphorylated IRS-1 and Akt in response to HCV infection. Etifoxine at 1 *μ*M of higher concentration reduced the IRS-1 and Akt phosphorylated levels in uninfected Huh7.5-SEAP cells (data not shown).

### 3.3. Pretreatment with Etifoxine Decreased the Insulin-Mediated Glucose Uptake under HCV Infection

**Figure 3:**
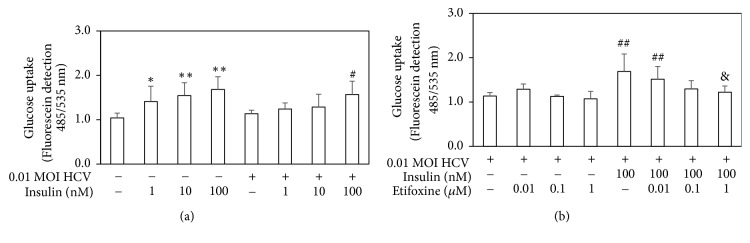
*Effects of etifoxine on the insulin-mediated glucose uptake under HCV infection.* (a and b) Huh7.5-SEAP cells (1 x 10^4^) were infected with 0.01 MOI of HCV and treated with etifoxine (0.01-1 *μ*M) or insulin (100 nM) in a 96-well plate. In some experiments, cells were treated with 0.01-1 *μ*M of etifoxine for 1 h before treatments of insulin. 6 days after treatment, cell culture media were replaced with glucose-free culture medium and incubated with 2-NBDG before the fluorescent detection at 485/535 nm. Data are expressed as mean ±SD obtained from three individual experiments. ^*∗*^*p* < 0.05 and ^*∗∗*^*p* < 0.01 vs. the medium control group; ^#^*p* < 0.05 and ^##^*p* < 0.01 vs. the HCV-infected Huh 7.5-SEAP group; ^&^*p* < 0.05 vs. the insulin-treated group.

The administration of TSPO ligands has been shown to influence glucose uptake [28, 29]. The effects of etifoxine on glucose uptake were therefore determined by using fluorescence glucose uptake 2-(N-(7-nitrobenz-2-oxa-1,3-diazol-4-yl)amino)-2-deoxyglucose (2-NBDG). As shown in Figure 3(a), the administration of insulin at 1-100 nM enhanced glucose uptake in naïve Huh7.5-SEAP cells. However, insulin-induced glucose uptake was only observed in HCV-infected cells treated with insulin at 100 nM, suggesting that HCV-induced IR. On the other hand, pretreatment with etifoxine worsened the insulin-mediated glucose uptake, whereas etifoxine alone did not affect this ability (Figure 3(b)).

### 3.4. The Insulin-Mediated IRS-1/Akt Signals and FoxO1 Translocation Were Blocked by Etifoxine Pretreatment in HCV Infection

**Figure 4:**
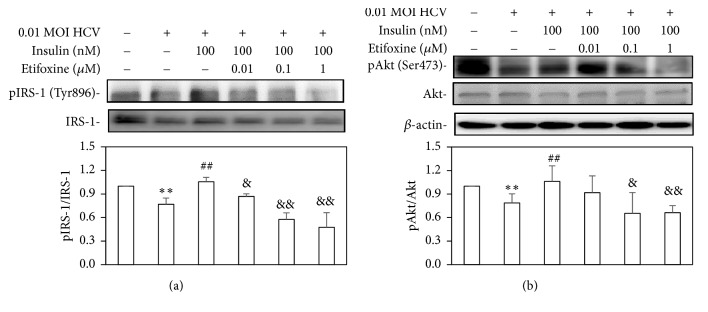
*Effects of etifoxine on the insulin-mediated IRS-1/Akt signals under HCV infection.* (a and b) Huh7.5-SEAP cells (7 x 10^5^) were infected with 0.01 MOI of HCV and treated with etifoxine (0.01-1 *μ*M) or insulin (100 nM) in a 6-well plate. In some experiments, cells were treated with 0.01-1 *μ*M of etifoxine for 1 h before treatments of insulin. Cell lysates were collected at day 6 and the phospho-IRS-1 (Tyr896), IRS-1, phospho-Akt (Ser473), Akt, and *β*-actin levels were detected by Western blot. Data are expressed as mean ±SD obtained from three individual experiments. ^*∗∗*^*p* < 0.01 vs. the medium control group; ^##^*p* < 0.01 vs. the HCV-infected Huh 7.5-SEAP group; ^&^*p* < 0.05 and ^&&^*p* < 0.01 vs. the insulin-treated group.

Administration of insulin at a concentration of 100 nM triggered the phosphorylation of IRS-1 on Tyr896 and Akt on Ser473. However, the insulin-mediated IRS-1 and Akt signals were reduced by etifoxine (Figures 4(a) and 4(b)).

**Figure 5:**
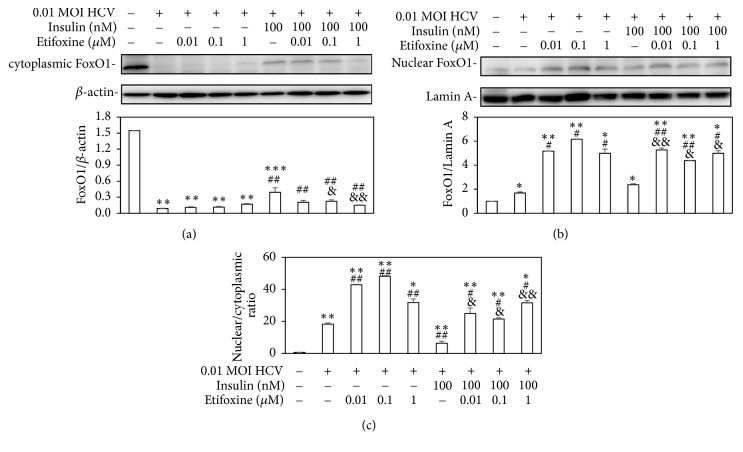
*Effects of etifoxine on the insulin-mediated FoxO1 expression under HCV infection.* (a and b) Huh7.5-SEAP cells (7 x 10^5^) were infected with 0.01 MOI of HCV and treated with etifoxine (0.01-1 *μ*M) or insulin (100 nM) in a 6-well plate. In some experiments, cells were treated with 0.01-1 *μ*M of etifoxine for 1 h before treatments of insulin. Cell lysates were collected at day 6 and the FoxO1, *β*-actin, and lamin A levels were detected by Western blot. (c) Nuclear/cytoplasmic ratio indicated the extent of FoxO1 translocation to nucleus. Data are expressed as mean ±SD obtained from three individual experiments. ^*∗*^*p* < 0.05, ^*∗∗*^*p* < 0.01, and ^*∗∗∗*^*p* < 0.001 vs. the medium control group; ^#^*p* < 0.05 and ^##^*p* < 0.01 vs. the HCV-infected Huh 7.5-SEAP group; ^&^*p* < 0.05 and ^&&^*p* < 0.01 vs. the insulin-treated group.

In addition, etifoxine induced FoxO1 translocation from cytoplasm (Figure 5(a)) to nucleus (Figure 5(b)) in the presence or absence of insulin. Nuclear/cytoplasmic ratio indicated the extent of FoxO1 translocation to nucleus and this is shown in Figure 5(c).

### 3.5. Etifoxine Decreased Lipid Accumulation via Downregulations of SREBP-1 and ApoJ

**Figure 6:**
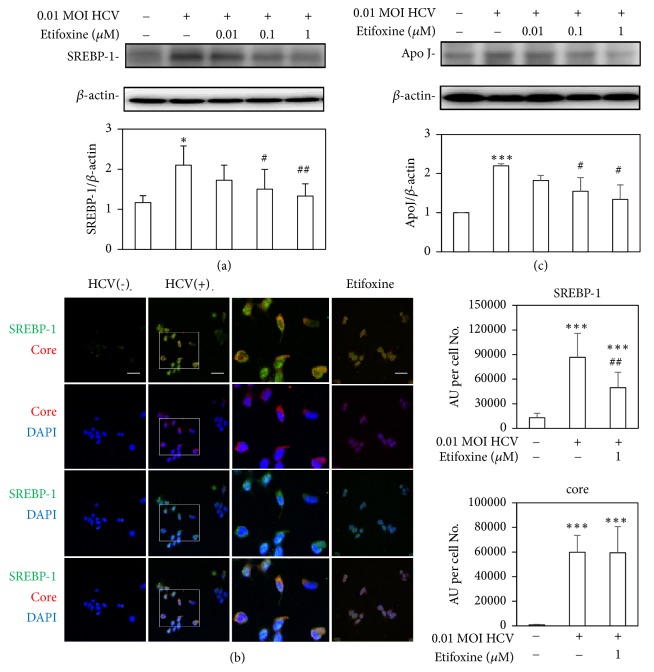
*Effects of etifoxine on SREBP-1 and apoJ protein levels.* (a and c) Huh7.5-SEAP cells (7 × 10^5^) were infected with 0.01 MOI of HCV and treated with etifoxine (0.01-1 *μ*M) in a 6-well plate for 6 days after treatment. Cell lysates were collected and the SREBP-1, apoJ, and *β*-actin levels were detected by Western blot. (b) Cells were infected with HCV and treated with 1 *μ*M of etifoxine for 6 days in a 24-well plate. Cells (1.5 x 10^4^) were fixed with paraformaldehyde on day 6, stained with core and SREBP-1 antibodies, counterstained with DAPI, and then observed under confocal laser scanning microscope. Scale bar: 50 *μ*M. The arbitrary units (AU) of immunofluorescence intensity normalized with the number of cells were assessed using Image J. Six fields per each group were randomly chosen and each field, containing approximately 15-20 cells, was amplified 40 fold in order to observe core protein expression and SREBP-1 translocation to nucleus. Data are expressed as mean ±SD obtained from three individual experiments. ^*∗*^*p* < 0.05 and ^*∗∗∗*^*p* < 0.001 vs. the medium control group; ^#^*p* < 0.05 and ^##^*p* < 0.01 vs. the HCV-infected Huh 7.5-SEAP group.

**Figure 7:**
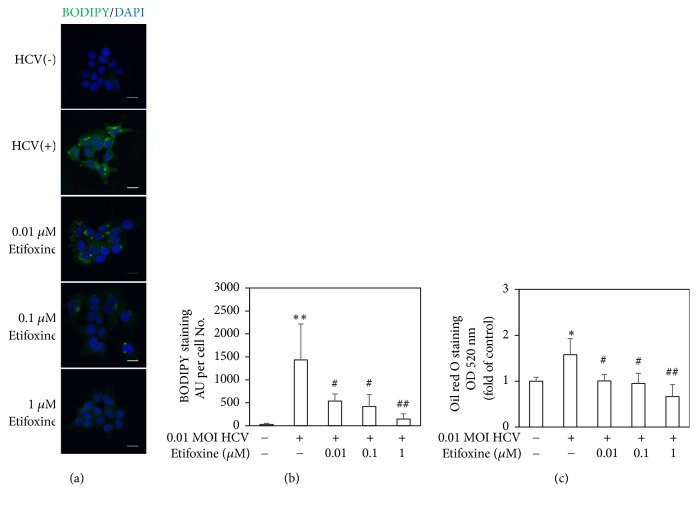
*Effect of etifoxine on lipid accumulation.* (a and b) Cells (4 × 10^3^) were infected with HCV and treated with 1 *μ*M of etifoxine for 6 days. Cells were fixed with paraformaldehyde on day 6, stained with neutral lipid dye BODIPY, counterstained with DAPI, and then observed under confocal laser scanning microscope. Scale bar: 20 *μ*M. The AU of immunofluorescence intensity normalized with the number of cells were assessed using Image J. Eight fields per each group were randomly chosen and each field, containing approximately 15-20 cells, was amplified 40 fold. (c) Cells (2 × 10^4^) were fixed with formaldehyde, stained with oil red O solution on day 6, added with IGEPAL-CA-630, and then determined by ELISA reader. Data are expressed as mean ±SD obtained from three individual experiments. ^*∗*^*p* < 0.05 and ^*∗∗*^*p* < 0.01 vs. the medium control group; ^#^*p* < 0.05 and ^##^*p* < 0.01 vs. the HCV-infected Huh 7.5-SEAP group.

SREBP-1 plays a crucial role in the regulation of gene expression of de novo lipogenesis and participates in HCV-induced IR [4]. HCV production depends on the assembly and secretion of apo; otherwise it seizes and interferes with host lipid metabolism [34–36]. In addition, TSPO ligands modulate lipid metabolism [11]. However, the effects of TSPO ligand on SREBP-1, apoJ, and lipid accumulation in HCV infection are still not fully understood. In our study, HCV-infected Huh7.5-SEAP cells augmented SREBP-1 protein expression (Figure 6(a)) and its translocation to the nucleus (Figure 6(b)), higher apoJ levels (Figure 6(c)), and lipid accumulation with BODIPY (Figures 7(a) and 7(b)) and oil red O staining (Figure 7(c)). On the other hand, etifoxine inhibited the HCV-induced SREBP-1 protein expression and blocked its translocation to the nucleus. Moreover, it also reduced lipid accumulation, accompanied with lower apoJ levels. These results indicated that SREBP-1 and apoJ may be involved in the pathogenesis of TSPO in HCV-induced lipid accumulation.

## 4. Discussion

**Figure 8:**
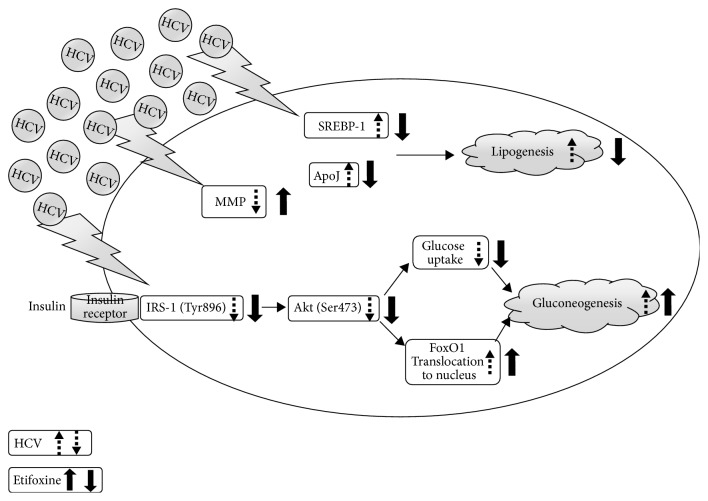
*A schematic model of the involvement of signal molecules in etifoxine-mediated IR and lipid metabolism under HCV infection.* Etifoxine itself rescued the HCV-mediated lower MMP, aggravated the HCV-impeded activations of IRS-1 and Akt, and inhibited the HCV-induced lipid accumulation accompanied with downregulation of SREBP-1 and apoJ. Alternatively, etifoxine blocked the insulin-mediated IRS-1/Akt signals, FoxO1 translocation, and glucose uptake. Dotted lines with arrows indicated the up- or downregulation of HCV-mediated signal molecules. Bold solid lines with arrows indicated the increased levels, decreased levels, or blockade of HCV-mediated signal molecules by etifoxine.

In the present study, we have first demonstrated that etifoxine (1) raised the TSPO protein expression and recovered the HCV-mediated lower MMP; (2) intensified the HCV-impeded activations of IRS-1 and Akt as well as HCV-induced nuclear translocation of FoxO1; (3) blocked the insulin-mediated IRS-1/Akt signals, FoxO1 translocation to cytoplasm, and glucose uptake; (4) inhibited the HCV-induced lipid accumulation, accompanied with the downregulation of SREBP-1 and apoJ. These results indicated that the activation of TSPO may partially reduce lipid accumulation with downregulations of SREBP-1 and apoJ but interfere with the insulin-mediated signal dysfunctions in HCV infection (Figure 8).

In agreement with previous studies [2–4, 36], this study also found that HCV-induced IR through several molecular pathways, including mitochondrial dysfunction (Figure 1(c)), blockades of IRS-1/Akt (Figures 2(a) and 4), and insulin-stimulated glucose uptake (Figure 3). Subsequently, HCV upregulated SREBP-1, a modulator for the lipogenesis and gluconeogenesis (Figures 6(a) and 6(b)). Moreover, HCV increased lipid accumulation along with the upregulation of apoJ (Figures 6(c) and 7).

FoxO1 is a controller of hepatic insulin sensitivity and the metabolism of glucose and lipid. And since insulin modifies the FoxO1 subcellular distribution via stimulating the translocation of FoxO1 outside the nucleus, it inhibits the expression of enzymes related to gluconeogenesis (i.e., phosphoenolpyruvate carboxykinase and glucose 6-phosphatase). However, HCV infection inhibits the insulin-stimulated translocation of FoxO1 from the nucleus to the cytoplasm, thus stimulating gluconeogenesis [37, 38]. Our findings were similar to the above previous studies [37, 38] and further indicated that etifoxine enhanced HCV-induced nuclear translocation of FoxO1 in the presence or absence of insulin (Figure 5).

Gatliff & Campanella infer that TSPO is considered as a transporter and its ligands are able to regulate mitochondrial function at nM concentrations [10]. Conversely, TSPO ligands at *μ*M concentrations have profound cellular effects in which TSPO is not required, including apoptosis [39], glucose homeostasis, and cellular energy production [28, 29]. We observed similar effects of etifoxine at the lower *μ*M concentrations on relieving the HCV-mediated lower MMP. Without inducing cytotoxicity, etifoxine at the same ranges of concentrations was able to induce a number of cellular responses, including IRS-1/Akt signal dysfunction, blockade of the insulin-stimulated glucose uptake, a rise of the FoxO1 translocation to the nucleus, downregulations of SREBP-1, and the alleviation of lipid accumulation in HCV infection.

On the other hand, HCV triggered abnormal porphyrin metabolism and heme synthesis pathway in hepatocytes [40]. TSPO also interacts with porphyrins and precursors of heme and its function has been related to porphyrin transport and heme biosynthesis. Furthermore, TSPO prevents accumulation of protoporphyrin IX through its catalytic activity and mediates porphyrin catabolism with the consumption of reactive oxygen species. Conversely, TSPO deficiency reduces the oxygen consumption rate and MMP. Thus, TSPO plays the role of an important guard against oxidative stress [14, 41–43]. These findings help us to interpret that etifoxine may partially relieve the HCV-downregulated MMP level through activation of TSPO.

It has been reported that TSPO ligands may have an indirectly partial effect on glucose metabolism despite the following results still being controversial. (1) PK 11195 (TSPO antagonist) reduces weight gain and glucose level of high-fat diet- (HFD-) induced obese mice, but they have a strong inductive effect on cytosolic phosphoenolpyruvate carboxykinase expression that stimulates gluconeogenesis [28]; (2) PK 11195 and FGIN-1-27 (TSPO agonist) mediates insulin-stimulated glucose uptake [29]; (3) Ro5-4864 (TSPO agonist) induces neuroactive steroids expression in the central nervous system, but it does not significantly modify body weight and blood glucose levels in streptozotocin- (STZ-) induced diabetic rats [44]; (4) AC-5216 (TSPO agonist) can reverse the higher plasma glucose and lower insulin in HFD-STZ rats [45]. In our study, etifoxine deteriorated the insulin signal pathway, which might worsen HCV-induced IR (Figures 2–5).

TSPO acts as an imaging biomarker for nonalcoholic fatty liver disease [7]. PK11195 improves hepatosteatosis through reducing inactive precursor of SREBP-1 protein levels in HFD-induced obese mice [28]. On the other hand, PK 11195, Ro5-4864, and FGIN-1-27 at *μ*M concentrations increase the mRNA expression of 3T3-L1 adipocytes differentiation marker Fabp4. However, both PK 11195 and FGIN-1-27 at 10 *μ*M downregulate the mRNA expression of lipogenesis markers (i.e., SREBP-1c and fatty acid synthase), whereas both ligands at 1 *μ*M upregulate the gene expression of lipolysis markers (i.e., hormone-sensitive lipase and adipose triglyceride lipase). Thus, TSPO may play diverse roles during adipogenesis and in differentiated adipocytes [29]. In our study, etifoxine at a lower *μ*M concentration inhibited the HCV-induced upregulation of SREBP-1 and apoJ, indicating that TSPO may improve HCV-induced lipid accumulation.

Etifoxine improves peripheral nerve regeneration and its functional recovery [46]. Etifoxine is a TSPO agonist, has an anxiolytic effect by enhancing neurosteroidogenesis, and is clinically approved for the treatment of anxiety disorders [46, 47]. However, etifoxine causes adverse drug reactions, including acute liver injury and cytolytic hepatitis with a possibly severe outcome [48, 49]. In our study, etifoxine partially rescued the HCV-disturbed MMP and lipid accumulation. However, it aggravated the HCV-induced insulin resistance by interfering with insulin signal molecules and did not directly modify HCV infection in the HCV-infected cells. Thus, the deterioration of HCV-induced insulin resistance by etifoxine in the long-term can be foreseen. Our study highlights the fact that great attention will be required in treating anxiety disorders with etifoxine in chronic hepatitis C patients with diabetes.

## 5. Conclusion

In our study, activation of TSPO by etifoxine rescued the HCV-mediated lower MMP and reduced lipid accumulation along with downregulations of SREBP-1 and apoJ, but it impeded insulin-mediated signal dysfunctions, including IRS-1/Akt signals, FoxO1 expression, and glucose uptake.

## Data Availability

The data used to support the findings of this study are available from the corresponding author upon reasonable request.

## Abbreviations

apo: Apolipoprotein
apoJ: Apolipoprotein J
AU: Arbitrary units
BODIPY: 4,4-difluoro-1,3,5,7,8-pentamethyl-4-bora-3a,4a-diaza-s-indacene
CCCP: Carbonyl cyanide 3-chlorophenylhydrazone
DAPI: 4,6-diamidino-2-phenylindole
FoxO1: Forkhead box protein O1
HCV: Hepatitis C virus
HFD: High-fat diet
IR: Insulin resistance
IRS: Insulin receptor substrate
MMP: Mitochondrial membrane potential
MOI: Multiplicity of infection
MTS: 3-(4,5-dimethylthiazol-2-yl)-5-(3-carboxymethoxyphenyl)-2-(4-sulfophenyl)-2H-tetrazolium
2-NBDG: 2-(N-(7-nitrobenz-2-oxa-1,3-diazol-4-yl)amino)-2-deoxyglucose
NS: Nonstructural protein
OD: Optical density
PBS: Phosphate buffered saline
RLU: Relative light unit
SD: Standard deviation
SEAP: Secreted alkaline phosphatase
SREBP: Sterol regulatory element-binding protein
STZ: Streptozotocin
TSPO: 18 kDa translocator protein.
